# 

**Published:** 1845-04

**Authors:** 


### CONTENTS OF Nº XXXVIII

OF THE

### British and Foreign Medical Review.

APRIL 1845.

---

### PART FIRST.—Analytical and Critical Reviews.

PAGE

ART. I.—Report of the Metropolitan Commissioners in Lunacy to the Lord Chancellor. Presented to both Houses of Parliament by command of Her Majesty . . . . . . . 317

ART. II.—A practical Treatise on the Diseases peculiar to Women, illustrated by Cases derived from Hospital and Private Practice. By SAMUEL ASHWELL, M.D. Member of the Royal College of Physicians, Obstetric Physician and Lecturer to Guy's Hospital . . . . . 344

ART. III.—Verhandlungen der K.K. Gesellschaft der Aerzte zu Wien . 360
Transactions of the Imperial Medical Association of Vienna. Vols. I, II.

ART. IV.—1. Hygiène Militaire, à l'usage des Armées de Terre. Par le CHEVALIER J. R. LOUIS DE KIRCKHOFF, &c. &c. . . . . 377

2. Vade-mecum ou Guide du Chirurgien Militaire. Par le CHEVALIER SARLANDIERE, D.M., &c. &c. . . . . . ib.

3. Manuel portatif des Officiers de Santé des Hôpitaux et des Corps de Troupes. Par A. DORAT . . . . . . ib.

4. Manuel réglementaire à l'usage des Officiers de Santé des Höpitaux Militaires et des Corps de troupes. Par J. A. A. E. PUEL, D.M. &c. &c. . ib.

5. Aide-mémoire Medico-legal de l'Officier de Santé de l'Armée de Terre; publié avec autorisation du Ministre de la Guerre, et encouragé par le conseil de Santé des Armées. Par F. C. MAILLOT, Professeur à l'hôpital militaire d'instruction de Metz; et J. A. A. E. PUEL, Médecin ordinaire, &c. . ib.

6. Elements d'Hygiène Militaire. Par D. PH. MUTEL, D.M. Chirurgien Major au 52e de ligne, &c. &c. . . . . . . ib.

7. On Military Hygiene, and particularly upon the Clothing of Soldiers. By FREDERICK ROBERTS, Esq. Assistant Surgeon, 59th Regiment . . ib.

ART. V.—Mémoires de la Société Médicale d'Observation de Paris . . 390
Memoirs of the Medical Society of Observation of Paris.

ART. VI.—1. Die Plastische Chirurgie nach ihren bisherigen Leistungen kritisch dargestellt. Eine von der Medicinischen Gesellschaft zu Gent gekrönte Preisschrift. Von Dr. FRIEDRICH AUGUST V. AMMON, Hofrath, Leibarzt S. M. des Königs von Sachsen, &c. &c., und Dr. MORITZ BAUMGARTEN, Practishem Arzte zu Dresden, &c. . . . . 396

Plastic Surgery, critically examined as to what it has hitherto accomplished; a Prize Essay of the Medical Society of Gent. By Dr. F. A. VON AMMON and Dr. MORITZ BAUMGARTEN.

PAGE

2. Traité sur l'Art de restaurer les Difformités de la Face, selon la Méthode par Déplacement, ou Méthode Française. Par M. SERRE, Professeur de Clinique chirurgicale à la Faculté de Médecine de Montpellier, &c. &c. . . 396

Treatise on the Art of Restoring Deformities of the Face, by displacement (sliding of the flap,) or the French method. By M. SERRE, Clinical Profes. at Montpellier, and Surgeon to the Hospital St. Eloi.

3. Cases of Deformities of various kinds, successfully treated by Plastic Operations. By THOMAS D. MÜTTER, M.D. Professor of Surgery in Jefferson Medical College, &c. . . . . . . ib.

4. Cases of Deformity from Burns, successfully treated by Plastic Operations. By T. D. MÜTTER, M.D. &c. . . . . . ib.

5. A Report on the Operations for Fissures of the Palatine Vault. By T. D. MÜTTER, M.D. &c. . . . . . . ib.

ART. VII.—A Practical Treatise on Midwifery. By M. CHAILLY, Doctor of Medicine, Professor of Midwifery, &c. &c. A work adopted by the Royal Council of Public Instruction. Translated from the French and Edited by GUNNING S. BEDFORD, A.M. M.D., Professor of Midwifery and the Diseases of Women and Children in the University of New York . . 416

2. A Practical Treatise on Midwifery; exhibiting the present advanced state of the Science. By F. J. MOREAU, Professor of Midwifery, &c. &c. Translated from the French by THOMAS FORREST BETTON; and edited by PAUL B. GODDARD, A.M. M.D., Lecturer on Anatomy, &c. &c. With Eighty Plates, comprising numerous separate illustrations . . . ib.

ART. VIII.—1. Instruction pratique sur le Magnétisme Animal. Par J. P. F. DELEUZE . . . . . . . 428

Practical Instruction on Animal Magnetism. By J. P. F. DELEUZE.

2. Du Magnétisme Animal en France, et des jugements qu'en ont portés les sociétés savantes, avec le texte des divers rapports faits en 1784 par les commissaires de l'Académie des Sciences, de la Faculté et de la Société Royale de Médecine, et une analyse des dernières séances de l'Académie Royale de Médecine, et du rapport de M. Husson; suivi de considérations sur l'apparition de l'Extase, dans les traitements magnétiques. Par ALEXANDER BERTRAND . . . . . . . . ib.

On Animal Magnetism in France, and the Decisions which Learned Societies have made with respect to it, with the Text of the different Reports made in 1784 by the commissioners of the Academy of Sciences, of the Faculty and of the Royal Society of Medicine; and an Analysis of the last Sessions of the Royal Academy of Medicine and of the Report of M. Husson; followed by Considerations on the Manifestation of Extacy in Magnetic Treatment. By A. BERTRAND.

3. Isis Revelata: An Inquiry into the Origin, Progress, and present State of Animal Magnetism. By J. C. COLQUHOUN, Esq. Advocate, F.R.S.E. . ib.

4. Untersuchungen über den Lebensmagnetismus und das Hellsehen. Von Dr. JOHAN CARL PASSAVANT . . . . . ib.

5. An Introduction to the Study of Animal Magnetism. By the Baron DUPOTET DE SENNEVOY . . . . . . . ib.

6. Histoire Académique du Magnétisme Animal, accompagnée de Notes et de Remarques critiques sur toutes les observations et expériences faites jusqu'à ce jour. Par C. BURDIN, Jeune, et FRED. DUBOIS (d'Amiens.) . . ib.

Academic History of Animal Magnetism, accompanied with Notes and Critical Remarks upon all the Observations and Experiments hitherto made. By C. BURDIN and F. DUBOIS.

7. Facts in Mesmerism, and Thoughts on its Causes and Uses. By CHARLES CALDWELL, M.D. . . . . . . ib.

8. The Zoist: A Journal of Cerebral Physiology and Mesmerism, and their applications to Human Welfare . . . . . ib.

PAGE

9. Neurypnology; or the Rationale of Nervous Sleep, considered in relation with Animal Magnetism. Illustrated by numerous Cases of its successful application in the Relief and Cure of Disease. By JAMES BRAID, M.R.C.S.E. M.W.S. &c. . . . . . . . 428

10. Manuel Pratique de Magnétisme Animal; exposition méthodique de procédés employés pour produire les phénomènes magnétiques, et leur application à l'étude et au traitement des maladies. Par ALPH. TESTE, Docteur en Médecine de la Faculté de Paris, Membre de plusieurs sociétés savantes 429

Practical Manual of Animal Magnetism, with a Methodical Exposition of the Processes employed to produce the Magnetic Phenomena, and their application to the Study and Treatment of Disease. By A. TESTE.

11. Facts in Mesmerism, with Reasons for a dispassionate Inquiry into it. By the Rev. CHAUNCY HARE TOWNSEND, A.M. late of Trinity Hall, Cambridge . ib.

12. Letters on Mesmerism. By HARRIET MARTINEAU . . . ib.

13. Medical Report of the Case of Miss H—— H——. By T. M. GREENHOW, Fellow of the Royal College of Surgeons of England, &c. . . ib.

14. Human Magnetism; its claims to dispassionate inquiry. Being an attempt to show the utility of its application for the relief of human suffering. By W. NEWNHAM, M.R.S.L. &c. . . . . . . ib.

ART. IX.—The Anatomy of the Arteries of the Human Body, and its applications to Pathology and Operative Surgery; with a series of Lithographic Drawings, the size of nature. By RICHARD QUAIN, F.R.S. . . . 486

ART. X.—Die männlichen und weiblichen Wollust-Organe des Menschen und einiger Säugetheire in anatomisch-pathologischer Beziehung untersucht und dargestellt. Von Dr. G. L. KOBELT, &c. . . . 507

The Male and Female Organs of Copulation in the Human Species and some Mammalia, examined and demonstrated in their Anatomical and Physiological Relations. By Dr. G. L. KOBELT, &c.

ART. XI.—Darstellung einer sicheren und schnellen Heilmethode der Syphilis durch Jodpräparate. Von Dr. GEORG MOJ'SISOVICS. . . . 516

An Account of a certain and rapid method of curing Syphilis by Preparations of Iodine. By Dr. G. MOJ'SISOVICS.

ART. XII.—The Principles of Surgery. By JAMES MILLER, F.R.S.E. . . 524

ART. XIII.—The Biographical Dictionary of the Society for the Diffusion of Useful Knowledge . . . . . . . . 530

ART. XIV.—Ueber ossificirende Schwämme oder Osteoid-geschwulste. Von JOH. MÜLLER: gelesen in der Hufelandschen med.-chirurg. Gesellschaft am 1 Sept. 1843. Müller's Archiv für Physiologie, &c. . . 537

On Ossifying Fungous Growths or Osteoid Tumours. By J. MÜLLER. Read at the Hufeland Medico-chirurgical Society on the 1st of Sept. 1843. In Müller's Archives for Physiology, &c.

ART. XV.—Memoir on the Sex of the Child as a Cause of Difficulty and Danger in Human Parturition. By J. Y. SIMPSON, M.D. . . . 541

ART. XVI.—De circumvolutionibus Funiculi umbilicalis fœtus vitæ haud raro infestis. Dissertatio inauguralis quam consensu et auctoritate gratiosi medicorum ordinis in Academia Heidelbergensi, proposuit G. A. MAYER, &c. . 546

Inaugural Dissertation on Circumvolutions of the Umbilical Cord not unfrequently endangering the Life of the Child. By Dr. G. A. MAYER, &c.

ATT. XVII.—A Bill for regulating the Profession of Physic and Surgery. Ordered by the House of Commons to be printed 25th Feb. 1845. . . 549

ART. XVIII.—A Bill enabling Her Majesty to grant New Charters to certain Colleges of Physicians and Surgeons. Ordered to be printed 25th Feb. 1845 . 552

ART. XIX.—Geology, Introductory, Descriptive, and Practical. By DAVID THOMAS ANSTED, M.A. F.R.S., &c. . . 554

### PART SECOND.—Bibliographical Notices.

PAGE

ART. I.—Prolegomeni sulle Neurosi, &c. &c. Memoria del Dottor Lorenzo Gemignani . . . . . . . 555
Prolegomena on the Neurosis, &c. &c. A Memoir by Dr. L. GEMIGNANI.

ART. II.—The Natural History of Animals; being the substance of Three Courses of Lectures, delivered before the Royal Institution of Great Britain. By THOMAS RYMER JONES, F.R.S., F.Z.S., &c. . . . . 556

ART. III.—The Philosophy of the Moving Powers of the Blood. By G. CALVERT HOLLAND, M.D. &c. . . . . . . . . 557

ART. IV.—Tausend Aphorismen über die Geburt des Menschen. Von JOSEPH HERMANN SCHMIDT, M.D. &c. . . . . . . ib.

A Thousand Aphorisms on the Birth of Man. By Dr. J. H. SCHMIDT.

ART. V.—Ueber die Verrichtung der Wurzeln des Rückenmarksnerven, &c. Von J. W. ARNOLD . . . . . . . 558
On the Functions of the Roots of the Spinal Nerves. Physiological and Pathological Researches for estimating the Doctrines of Bell. By J. W. ARNOLD.

ART. VI.—Des Abscès Phlegmomeneux Intra-Pelviens. Par M. MARCHAL (DE CALVI) . . . . . . . . . 559
On Intra-Pelvic Phlegmonous Abscesses. By M. MARCHAL (DE CALVI).

ART. VII.—A Treatise on Corns, Bunions, the Diseases of the Nails, and the general management of the Feet. By LEWIS DURLACHER, Surgeon Chiropodist (by special appointment) to the Queen . . . . . 560

ART. VIII.—Practical Observations and Suggestions on Medicine. By MARSHALL HALL, M.D. F.R.S.L. & E. &c. &c. . . . . . . 561

ART. IX.—A Thermometrical Table on the Scales of Fahrenheit, Centigrade, and Reaumur; comprising the most remarkable phenomena connected with Temperature in relation to Climatology, Physical Geography, Chemistry and Physiology. By ALFRED S. TAYLOR, Lecturer on Chemistry, &c. . 562

---

### PART THIRD.—Original Reports and Memoirs.

1. Report on the Progress of Human Anatomy and Physiology, in the years 1843-4. Part II. By JAMES PAGET . . . . . . . 563

2. Notices of the Lunatic Asylums of Paris. In a Letter to John Forbes, M.D.F.R.S. By JOHN CONOLLY, M.D., Fellow of the Royal College of Physicians, Physician to the County Lunatic Asylum at Hanwell . . . 594

### INTELLIGENCE.

1. Schools for the Sons of Medical Men . . . . . . 613
2. National Association of General Practitioners . . . . . 614
3. Books received for review . . . . . . . 618
INDEX, TITLE, &c. &c.

